# Implementation of a diabetes in pregnancy clinical register in a complex setting: Findings from a process evaluation

**DOI:** 10.1371/journal.pone.0179487

**Published:** 2017-08-04

**Authors:** Renae Kirkham, Cherie Whitbread, Christine Connors, Elizabeth Moore, Jacqueline A. Boyle, Richa Richa, Federica Barzi, Shu Li, Michelle Dowden, Jeremy Oats, Chrissie Inglis, Margaret Cotter, Harold D. McIntyre, Marie Kirkwood, Paula Van Dokkum, Stacey Svenson, Paul Zimmet, Jonathan E. Shaw, Kerin O’Dea, Alex Brown, Louise Maple-Brown, on behalf of the Northern Territory Diabetes in Pregnancy Partnership

**Affiliations:** 1 Wellbeing and Preventable Chronic Disease Division, Menzies School of Health Research, Darwin, Australia; 2 Royal Darwin Hospital, Darwin, Australia; 3 Top End Health Service, Northern Territory Department of Health, Darwin, Australia; 4 Aboriginal Medical Services Alliance Northern Territory, Darwin, Australia; 5 Monash Centre for Health Research and Implementation, School of Public Health and Preventive Medicine, Monash University, Melbourne, Australia; 6 Health Gains Planning Branch, Department of Health, Northern Territory Government, Darwin, Australia; 7 Health Services and Planning, Sunrise Health Service Aboriginal Corporation, Katherine, Australia; 8 Melbourne School of Population and Global Health, University of Melbourne, Melbourne, Australia; 9 Healthy Living Northern Territory, Darwin, Australia; 10 Mater Medical Research Institute, University of Queensland, Brisbane, Australia; 11 Alice Springs Hospital, Northern Territory, Alice Springs, Australia; 12 Population Health Research, Baker IDI Heart and Diabetes Institute, Alice Springs, Australia; 13 School of Biomedical Sciences, Monash University, Melbourne, Australia; 14 Population Health Research, Baker IDI Heart and Diabetes Institute, Melbourne, Australia; 15 Population Health Research, University of South Australia, Adelaide, Australia; 16 South Australian Health and Medical Research Institute, Adelaide, Australia; Coventry University, UNITED KINGDOM

## Abstract

**Background:**

Rates of diabetes in pregnancy are disproportionately higher among Aboriginal than non-Aboriginal women in Australia. Additional challenges are posed by the context of Aboriginal health including remoteness and disadvantage. A clinical register was established in 2011 to improve care coordination, and as an epidemiological and quality assurance tool. This paper presents results from a process evaluation identifying what worked well, persisting challenges and opportunities for improvement.

**Methods:**

Clinical register data were compared to the Northern Territory Midwives Data Collection. A cross-sectional survey of 113 health professionals across the region was also conducted in 2016 to assess use and value of the register; and five focus groups (49 healthcare professionals) documented improvements to models of care.

**Results:**

From January 2012 to December 2015, 1,410 women were referred to the register, 48% of whom were Aboriginal. In 2014, women on the register represented 75% of those on the Midwives Data Collection for Aboriginal women with gestational diabetes and 100% for Aboriginal women with pre-existing diabetes. Since commencement of the register, an 80% increase in reported prevalence of gestational diabetes among Aboriginal women in the Midwives Data Collection occurred (2011–2013), prior to adoption of new diagnostic criteria (2014). As most women met both diagnostic criteria (81% in 2012 and 74% in 2015) it is unlikely that the changes in criteria contributed to this increase. Over half (57%) of survey respondents reported improvement in knowledge of the epidemiology of diabetes in pregnancy since establishment of the register. However, only 32% of survey respondents thought that the register improved care-coordination. The need for improved integration and awareness to increase use was also highlighted.

**Conclusion:**

Although the register has not been reported to improve care coordination, it has contributed to increased reported prevalence of gestational diabetes among high risk Aboriginal women, in a routinely collected jurisdiction-wide pregnancy dataset. It has therefore contributed to an improved understanding of epidemiology and disease burden and may in future contribute to improved management and outcomes. Regions with similar challenges in context and high risk populations for diabetes in pregnancy may benefit from this experience of implementing a register.

## Introduction

The prevalence of diabetes continues to increase globally. Diabetes in pregnancy (DIP) provides an opportunity to intervene early in the life course for mother and child. Pregnancies complicated by diabetes (pre-existing and gestational diabetes) pose a challenge for management, as timely diagnosis and implementation of best-practice care have implications for both maternal and foetal outcomes [1–3].

In Australia, the prevalence of type 2 diabetes in pregnancy and gestational diabetes mellitus (GDM) is higher (10 and 1.5 times respectively) for Aboriginal and Torres Strait Islander Aboriginal) women than non-Aboriginal women [4]. We respectfully acknowledge the two Indigenous populations of Australia, the Aboriginal and Torres Strait Islander people, who are referred to as Aboriginal people in this paper. In 2013, 31% of all births in the Northern Territory (NT) were to Aboriginal mothers, with available data reporting higher rates of GDM among Aboriginal than non-Aboriginal women (16% vs 10% respectively) [5]. The NT covers a large geographical area of 1.35 million square kilometres but has a relatively small population (245,100). Aboriginal people make up 29.6% of the total NT population and 80% of Aboriginal people live in non-metropolitan and remote areas [6].

Changes in GDM diagnostic guidelines occurred both in Australia and internationally during the course of this study. Between 2012–2014 there was a gradual increase in implementation of new guidelines in the NT where women with GDM were diagnosed by either the Australian Diabetes in Pregnancy Society (ADIPS) [7] guidelines (of Glucose Challenge Test then Oral Glucose Tolerance Test) or a universal 75gm Oral Glucose Tolerance Test (as recommended by International Association of the Diabetes and Pregnancy Study Groups (IADPSG) [8] and the World Health Organisation (WHO) [9]). The WHO guidelines were formally adopted from 1 January 2014. The glucose diagnostic cut-points also changed (from ≥ 5.5mmol/L fasting, ≥7.8 mmol/L 2-hrs (Alice Springs Hospital) and ≥8.0mmol/L 2-hrs (all other hospitals) to ≥ 5.1mmol/L fasting, ≥ 10 mmol/L 1-hr and ≥ 8.5mmol/L 2-hrs [10]).

A DIP Clinical Register was established in 2011 by the NT DIP Partnership to address the gap in knowledge regarding the prevalence of DIP in the NT, and to aid in care coordination of this condition in the context of challenges experienced in Aboriginal health. In this region, these challenges include remote residence, socio-economic disadvantage and a high risk population [11,12].

Clinical registers (CR) are a systematic collection of a clearly defined set of data for patients with a specific health condition; and have been utilised to improve clinical outcomes in a number of settings [13–15]. They are utilised for many reasons, from monitoring effectiveness and safety to describing practice patterns, measuring outcomes, facilitating surveillance and benchmarking performance; thus providing structural frameworks for high quality care [16–18]. Progress in information technology and increasing demands for accountability have led to an increase in the number of registers over recent years [19].

The DIP CR was established with aims to: (i) improve the management of women with DIP by assisting improved care coordination and centrally collating key information (between primary and tertiary systems) to assist communication between providers; (ii) improve follow-up of women with DIP; (iii) act as a quality assurance tool; and, (iv) act as an epidemiological tool to establish the DIP burden and its variability over time, place and ethnicity. See Supporting Information for detailed information about the NT DIP Partnership, Governance Structure and additional CR methods.

The aim of this manuscript is to identify the successes of implementing this CR, the ongoing challenges and opportunities for improvements.

## Methods

A mixed methods approach was selected to triangulate the internal validity of multiple sources of data; the Clinical Register, Midwives Data Collection, Health Professional Survey and Focus Groups.

### Ethics approval

Ethics approval for this work was obtained from the Human Research and Ethics Committee of the NT Department of Health and Menzies School of Health Research and the Central Australian Human Research and Ethics Committee.

### Clinical register

All women residing in NT of age 16 years and above with any type of DIP (type 1, type 2 and gestational diabetes) are eligible for the Clinical Register. Participants gave informed verbal consent to be included on the Clinical Register. Consent was obtained by their referring health practitioner who completed a written form at the time of referral. This form is consistent with the Operating principles of the Australian Commission on Safety and Quality in Health Care’s Framework for Australian clinical quality registries [18]. Information collected is forwarded to CR managers who are trained in data entry and management. Health professionals can apply for access to the CR for read-only purposes via an online password-secured link. Associated documents such as referral forms, operating principles and information forms are also located online [20] (see supplementary materials for details of Clinical Register).

Clinical Register reports include a summary of key findings including maternal characteristics, type of diabetes and birth outcomes. Reports are stratified into two main regions of the NT and summary data for smaller jurisdictions can be collated and supplied on request. Clinical Register meetings are held with key stakeholders biannually to review the reports, discuss implications for clinical practice and current processes, identify key issues for improvement and engage relevant stakeholders to implement change.

### Comparisons to Northern Territory Midwives Data Collection

Data from the CR was compared to available NT Midwives Data Collection (MDC) (2010–2014 [21–24]). Although data from 2014 has not been published, it has made available from NT Perinatal data for purposes of this manuscript. Approval to publish this data was obtained from the Health Gains Planning Branch, Department of Health, Northern Territory Government. It is a mandatory requirement that midwives enter birth data for all NT births into the MDC. The data set provides annual reports on population characteristics and birth outcomes of mothers who give birth in the NT. Note that CR data have been shared on an annual basis with MDC such that CR data are used to validate MDC DIP data, particularly for pre-existing diabetes.

### Survey

A cross-sectional survey of health professionals was performed to evaluate CR use. The survey contained 32 questions covering four themes: knowledge, use, value and improvements. Participants included all health professionals involved in antenatal care and were contacted from May to August 2016 through email using health professional networks (government and non-government, primary healthcare including Aboriginal Community Controlled Health Organisations and hospital care) and at regional meetings and forums. Users were defined as respondents who reported use of the register. Respondents could determine their use of the CR to be primarily web-based for individual patient care (identified data) and/or report based for quality assurance (de-identified grouped data).

### Focus groups

A recent interim evaluation of the Partnership involved six focus groups with 49 health professionals directly involved in DIP care from across the NT (October 2015 –February 2016) to obtain an understanding of the enablers and persisting barriers to models of care. This qualitative work was underpinned by a phenomenological methodology [25]. The Systems Assessment Tool [26,27] guided discussions to gain insight into health professionals’ experiences and understandings of factors influencing the strengths and weaknesses of the health system. These included: delivery system design; information systems and decision support; self-management support; links with community and other services; and organisational influence and integration. Participants were recruited through Partnership networks, informed consent obtained and focus groups conducted by facilitators. Data were audio recorded, transcribed and deductively analysed in line with the components of the Systems Assessment Tool (as listed above). Coding structures were cross-checked for accuracy by RK and MD. Results pertaining to the CR are reported in this manuscript and compliment findings from the Health Professional Survey.

### Statistical methods

Data were analysed using the statistical software STATA 14.2. Mean values were reported for age and simple frequencies and percentages for all the other variables.

## Results

### Clinical register results

#### i. Prevalence of diabetes in pregnancy

From January 2012 to December 2015, 1,410 women with DIP were referred to the CR, of whom 673 (48%) were Aboriginal women. Overall, 83% of women had GDM and 15% type 2 diabetes (of whom 86% were Aboriginal). There have not been any withdrawals from the CR to date (December 2015). Aboriginal women were younger (29 v 31 years) and more likely to live in regional or remote areas (75% v 9%) than non-Aboriginal women.

**Fig 1:**
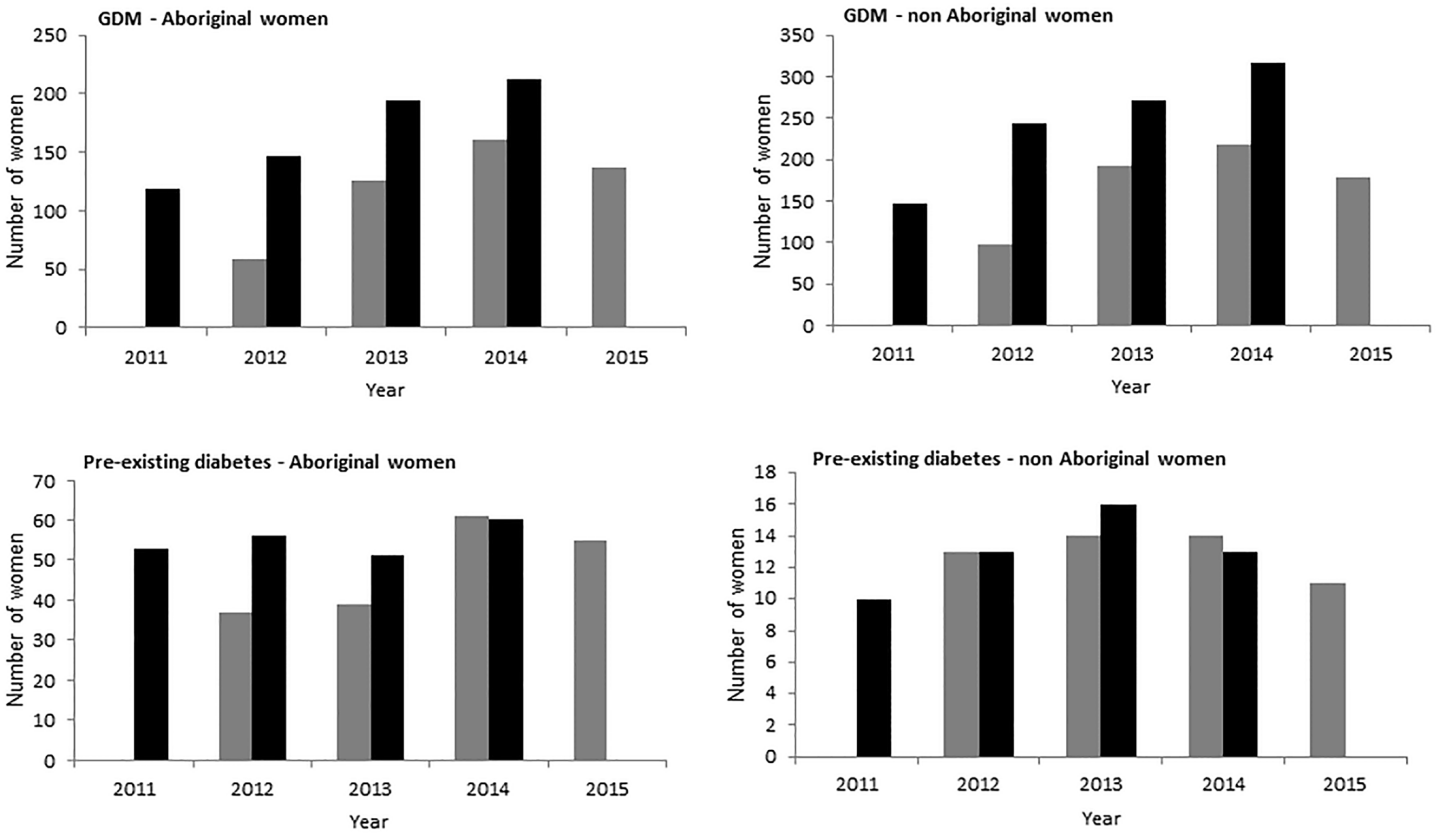
Numbers of GDM and pre-existing diabetes in the NT as reported by NT Midwives Data Collection as compared to NT DIP Clinical Register. MDC did not report on pre-existing diabetes in 2010 and data not yet published 2015; Pre-existing diabetes includes Type 1. Total number of births on MDC for 201, 2012, 2013 and 2014 were as follows: 2011- Aboriginal *n* = 1349, non-Aboriginal *n = 2440*, 2012- Aboriginal *n* = 1348, non-Aboriginal *n* = 2556, 2013- Aboriginal *n* = 1232, non-Aboriginal *n* = 2687, and 2014- Aboriginal *n* = 1315, non-Aboriginal *n* = 2610. Based on these total births the prevalence of GDM among all pregnancies in Aboriginal women was 8.7% in 2011 and 15.7% in 2013; in non-Aboriginal women it was 6.0% in 2011 and 10.1% in 2013. The prevalence of pre-existing diabetes among all pregnancies in Aboriginal women was 3.9% in 2011 and 4.1% in 2013; in non-Aboriginal women it was 0.4% in 2011 and 0.6% in 2013.

The number of women with any DIP recorded on the CR increased each year from baseline until plateauing in year 4. The number of women with DIP on the CR as a proportion of those recorded with DIP on the MDC also increased over the four years of the register (Fig 1). Only data up to and including year 2014 are currently available for the midwives data collection. Thirty-nine percent of Aboriginal women with GDM were on the CR in 2012 (58/147) which increased to 65% (126/194) in 2013 and 75% (160/212) in 2014. Sixty-six percent of Aboriginal women with pre-existing diabetes were on the CR in 2012 (37/56) which increased to 76% (39/51) in 2013 and over 100% (61/60) in 2014. Furthermore, numbers of women with DIP in the MDC have increased significantly during the time period of the DIP Partnership and CR such that there was an 80% increase ({[15.7–8.7]/8.7}×100 (Fig 1)) in reported prevalence of GDM among Aboriginal women reported on the MDC (2011–2013), prior to adoption of new diagnostic criteria (2014). Known data not included in the CR are of women who birthed in 2 regional and 1 private urban hospital (total births in those hospitals = 1,099 of total 4,018 NT births in 2013 [5]). Increasing proportions of women on the CR were diagnosed by new GDM criteria in 2014–2015 (which were introduced as policy change in 2014); with a greater increase among non-Aboriginal women. Rates of diagnosis by both criteria remained relatively stable (Table 1).

**Table 1:** Proportion of women with GDM on CR by diagnostic criteria *n*(*%)*.

	IADPSG [8] /WHO [9]			IADPSG, WHO and ADIPS [7]		
	All	Non-Indigenous Women	Indigenous Women	All	Non-Indigenous Women	Indigenous Women
**2012**	2 (1)	1 (1)	1 (2)	120 (81)	74 (79)	46 (85)
**2013**	8 (3)	4 (2)	4 (4)	236 (77)	146 (78)	90 (76)
**2014**	49 (13)	34 (16)	15 (9)	294 (80)	166 (77)	128 (85)
**2015**	66 (22)	44 (27)	22 (17)	219 (74)	115 (70)	104 (79)

### Use of clinical register

Of the 62 health care professionals with web-based access to the read-only CR (including midwives, diabetes educators, endocrinologists, obstetricians and Aboriginal Health Practitioners), 18 have accessed it 188 times to date (December 2015), including; 7 people in 2013 (1 midwife and 6 diabetes educators); 12 people in 2014 (3 midwives and 9 diabetes educators); and 10 people in 2015 (including 5 midwives and 5 diabetes educators). CR reports (de-identified grouped data summary statistics) have been distributed to clinicians since 2013.

### Survey results

#### i. Participants

**Fig 2:**
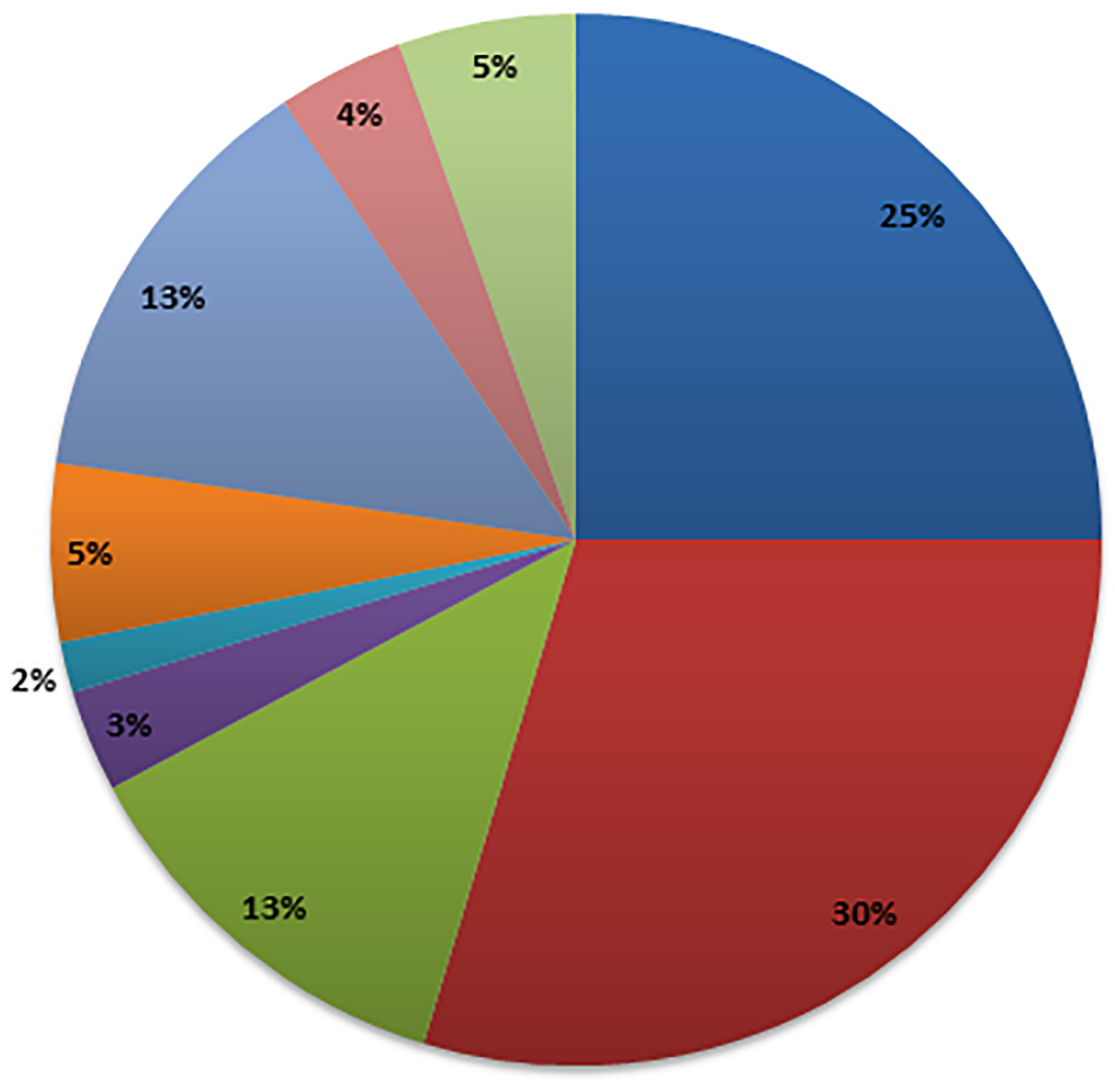
Occupation of survey respondents. Total *n* = 113: of the 38 registered midwives, 17 were also registered nurses; 4 diabetes educators were registered midwives; 1 dietician was also a diabetes educator; 1 Aboriginal Health Practitioner was also a diabetes educator.

One hundred and thirteen healthcare professionals from across the NT completed the survey, predominantly registered midwives, diabetes educators, general practitioners and registered nurses who are associated with services responsible for managing pregnant women (Fig 2). Forty-four percent of the 45 NT members of the Australian Diabetes Educators Association responded to the survey. Fifty-five percent of all respondents were living in an urban locality, 39% remote and 6% regional. Ninety percent of respondents were between the ages of 30–59. Fifty eight percent of respondents were working in a primary health centre (including government and Aboriginal Community Controlled Health Organisations), 32% in a hospital and 9% in general practice.

#### ii. Current clinical register use

The majority (67%, *n* = 76) of respondents had heard of the CR, of whom 21(28%) had reported using it more than once. Information on use was missing for two participants. Table 2 outlines differences between users and non-users: there were more diabetes nurse educators in the user group and no difference in time in position. Of the 53 respondents who gave a response to what type of information they have been provided with, 23 reported either the purpose, access or how to register for the CR. Only 37% of users reported knowing how to refer a woman to the CR with the main methods of referral being hand-filled paper referral (26%), electronic referral, email and fax (45%) and through professional networks (29%). The majority (84%, *n* = 75) of non-users reported not knowing how to refer.

**Table 2:** Users vs non-users.

	User *n(%)*	Non-user *n(%)*	*p value*
**Total Health Profession**	**21(19)**	**90(81)**	
Diabetes Educator	11(52)	7(8)	0.089
Registered Midwife	8(38)	34(38)	1.00
Registered Nurse	6(29)	28(31)	0.86
General Practitioner	-	17(19)	N/A
Endocrinologist	2(10)	2(2)	0.073
Aboriginal Health Practitioner	2(10)	3(3)	0.16
Other	2(10)	2(10)	0.77
Obstetrician	1(5)	1(1)	0.21
Other Medical Practitioner	1(5)	6(7)	0.74
Dietician	-	9(10)	N/A
**Time in job**			
>2 years	13(62)	43(48)	0.25
<2 years	7(34)	42(47)	
**Know how to apply for access**	16(76)	14(16)	<0.001
**Purpose of register has been outlined**	17(81)	33(37)	<0.001
**Heard of register**	21(100)	53(59)	<0.001
**Information provided to them**	20(95)	37(41)	<0.001
**Information considered useful**			
Past obstetric history	16(76)	62(69)	0.53
Current management	19(90)	80(89)	0.89
Latest clinical review	17(81)	76(84)	0.74

Note: Data on use was missing for two participants, thus the total sample here is 111.

Of participants who reported using the CR, 63% reported it was useful in assisting with client management. There was no significant difference between users and non-users in terms of what information they considered useful (Table 2). Of the 20 users (missing data *n* = 1), 80% (*n* = 16) reported the Partnership has improved education, orientation and guidelines, 70% (*n* = 14) reported the CR as being easy to access, and 60% (*n* = 12) reported an improvement of communication between sectors and services involved in DIP since the commencement of the CR and Partnership in 2012. Answers to open-ended questions and complementary extracts from focus groups that describe the CR in terms of its role and future improvements are detailed in Table 3.

**Table 3:** Open ended survey responses and focus group data.

*Themes*	*Survey Responses*	*Focus Groups*
***Role of CR***		
*Care coordination*	To improve medical outcomes of the women with DIP by assisting in care coordination for the women by collecting clinical information with the women’s consent and share the information with care providers (i.e. at primary level, specialist, educators—diabetic, nutritionist). Register has this info to assist and ensure the women are followed up.	*‘[It has] helped with systematic follow-up*, *because you have your lists*.’ [Endocrinologist]
	Provide a clear and easily accessible and integrated clinical record, decision support and links to relevant reference information and easy access to pathology, radiology and specialist letters.	*‘[The implementation of the clinical register] has been an important step forward*, *because you can’t do good chronic disease management if you don’t have a disease register*. *You need a mechanism for review*.*’* [Public Health Physician]
*Communication*	A tool to enable multiple clinicians in different roles and different sites to access updated care plans and medication doses for individual women leading to improved communication and quality of care.	*‘I am very mindful that it is there and very glad that it is there and would hope that over time it is something that is going to be informing our practice and probably streamline information*.*’* [Midwife]
	To review clinical pathways within multidisciplinary team.	
*Education*	Better care coordination for clients in remote setting.	*‘[It provides information about the prevalence of DIP so health professionals] can forward plan*, *and they can prompt their colleagues across the whole [region] in terms of following up*. *So that’s been a huge step forward*.*’* [Public Health Physician]
	Informing practitioners about clients’ attendance at DANCE clinic, allowing services/support workers such as Aboriginal Liaison workers to concentrate on supporting attendance of non-engaged/poorly attending clients.	
	Excellent information regarding DIP for women involved in our service. Encourages education and support.	
***Improvements***		
*Access*, *Integration*, *Promotion*	Be more accessible to NGO’s in education, promoting the register and in-services. Come to clinics.	*‘As soon as women come in [to clinic]*, *the next day we can see adjustments and increments and that stuff has been done*.*’* [Midwife]
	Include in induction to new staff or other ways to ensure people are aware of it and its relevance to them.	*‘… in the longer term the register will have huge inroads for feeding back information*, *but it is early days for the register really isn’t it*. *In the bigger scheme of data collection*.*’* [Midwife]
	A recall system, ability for end users to add information.	
	The register should be integrated into existing electronic records rather than a standalone for it to be of any use other than a research tool.	
	Enter data in real time and improve recruitment to the register	
***Other comments***		
*Useful*, *Don’t know about it*, *Not being used*	I have never used it to look up an individual woman but think it’s very useful for grouped reports.	Meetings and reports are thought to *‘increase regional capacity*.*’* [Diabetes Educator]
	Is a great collection of women with DIP but not accessed for guidance in how to manage [client] in collaboration with other care providers.	
	The CR is not being used to its full potential yet.	
	I don’t know about the register. Need to provide details of what it is, how to access it and how to register patients.	
	Don’t need another register and password to remember.	

#### iii. Improvements attributed to clinical register

**Fig 3:**
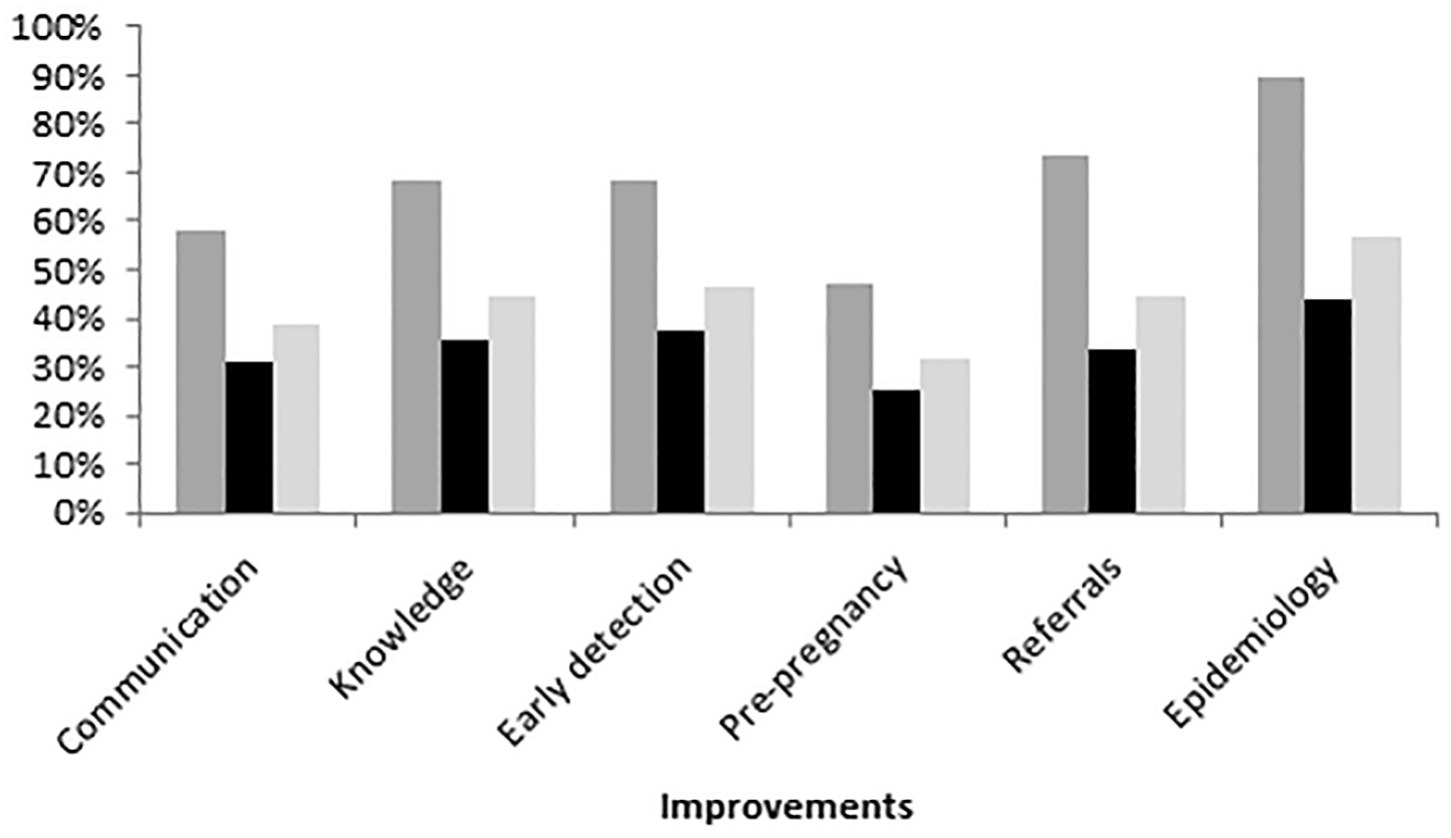
Improvements attributed to the clinical register. Communication: Increased communication; Knowledge: Improved understanding of the recommended clinical care required for women with DIP; Early detection: Improved awareness of early detection of DIP; Pre-pregnancy: Improved awareness of pre-pregnancy planning & contraception; Referrals: Improved awareness of who to contact in regards to women with DIP; Epidemiology: Improved awareness of how many women in the NT have DIP.

Seventy-four percent of all respondents believed that CRs are useful in assisting individual clinical care for women with DIP. Improvements attributed to the CR by survey participants included (Fig 3): awareness of the epidemiology of DIP in the NT (57%), awareness of clinical referral processes (45%), awareness of early detection of DIP (46%), and understanding of recommended clinical care (45%). Rates were higher within groups of users than non-users (e.g. 89% vs 44% for epidemiology, respectively).

#### iv. Care coordination

Only thirty-two percent (*n* = 32) of respondents believed care-coordination had improved since 2012 with the implementation of the CR (58% of these are users of the CR). Reasons given included improved access to clinical information, communication and increased confidence in managing clients. Of the 12% (*n* = 12) who did not think care coordination had improved, 42% (*n* = 8) were users of the CR, with reasons including its low visibility and minimal usage. Fifty-six percent of respondents were unsure if there had been improvements.

#### v. Reports and meetings

Of the 26 respondents who reported having received at least one CR report, the majority (61%) reported these as useful. Sixty-five percent of participants who had received a report were also users of the register. Of the 27 respondents who attended regional meetings, 25 found them useful. For those who had not received reports or been involved in meetings, most indicated they would like to receive reports (80%), and 50% reported wanting to attend meetings.

### Focus group data

Comments made about the CR were positive and related to its usefulness, improvements to care, care-coordination and integration (see Table 3 for a summary of themes). Specifically, they suggested that the establishment and implementation of the CR has improved epidemiology and communication between relevant stakeholders.

## Discussion

This manuscript aimed to assess the implementation of a clinical register. A mixed methods approach was used to consolidate findings around the successes, enduring challenges and opportunities for improvement. We report three key findings around its establishment and first four years of use, including: use as an epidemiological and quality assurance tool, limited use in individual patient care; and, opportunities for improved integration and promotion of the CR.

### Epidemiological and quality assurance

Consistent findings from clinicians in both the survey and focus groups were that perceived strengths and utility of the CR relate to two of the four originally stated aims: the use of CR as an epidemiological and quality assurance tool. Both have been reported as important in improving the quality of patient care [28]. The CR has strengthened the MDC as an epidemiological tool by likely contributing to an increased prevalence, as reported by MDC; thereby contributing to a better understanding of DIP burden of disease, which will likely improve early pregnancy testing among the high risk Aboriginal population and assist in improved management and outcomes in the future. Data from 2014 is difficult to compare to previous years because of the change in diagnostic criteria however data captured by the CR in comparison to the MDC, indicates improved coverage of the CR over several years. A similar increased understanding of disease prevalence was demonstrated in an evaluation of a CR to improve diabetes care in other Australian Aboriginal communities [15]. It is notable that numbers reported in the MDC increased significantly during the initial years of the Partnership and CR. Contributing factors may include increased awareness, screening and reporting of DIP generated by the CR and likely also related to work of the Partnership. It is unlikely that the changes in glucose diagnostic cut-points contributed to the increase as most women met diagnostic criteria for both the previous and current guidelines (81% in 2012 and 74% in 2015 on the CR) and the reported increase occurred prior to formal policy change of adoption of new criteria. It is of interest that with new GDM criteria alone, the proportion of Aboriginal women fell relative to non-Aboriginal women. This is consistent with a previous study in Far North Queensland [29]. An additional change that may have impacted prevalence is the change in diagnostic process (from two-step to one-step, which has previously been reported to result in increased prevalence without a change in cut-points [30]). It is notable that the coverage for those with pre-existing diabetes by the CR is 100% if MDC data. This could perhaps be attributed to significant work of the Partnership to increase clinicians’ understanding of the greater risk associated with pre-existing diabetes.

As a quality assurance tool, the CR was reported by users to have contributed to improvements in quality and efficiency of DIP management (including communication, early detection and referrals of women with DIP). However, the number of users in the survey was small and non-users did not report these changes. This is consistent with findings of a study reviewing rheumatic heart disease register programs, reporting the importance of the local context in establishing registers [14]. Furthermore, as quality assurance tools, CR’s benefit from appropriate dissemination of data which can be strengthened by governance structures and are important in informing policy [31]. The Partnership is well placed to disseminate relevant information, including reports, through its governance structure and networks.

### Individual patient care

The second finding is that despite most survey respondents reporting that CRs are useful in assisting with providing individual care, this was not reflected in usage. Some acknowledged its potential utility in relation to this aim, however reasons for not using it included a lack of awareness and integration. Sixty-eight per cent thought the CR did not improve care-coordination. This may be related to inter current activities of the Partnership addressing that issue (including educational workshops, forums and reports). These activities involved clinicians, service delivery providers and policy makers and focused on ways to improve patient-centred care and uptake of evidence-based practice. It is likely that this contributed to improved communication between sectors and disciplines, with the CR no longer required for that purpose. While there is some discrepancy between survey results (with more people having indicated they use it than those registered for web-based use), this suggests that reported use is broader than log-in use for individual patient-care and likely includes use of reports and/or discussion of reports at meetings. Providing health care professionals with feedback improves their motivation to use a register and the quality of data collected can be improved [17]. The Partnership has been consulting and feeding back to health professionals since 2011 and will continue to provide feedback to established networks and partnerships, including further discussion as to whether the aims of the CR should continue to include that of individual patient-care in addition to reports.

### Opportunities for improvement

Further opportunities for integration and promotion of the CR to all health professionals involved in DIP were identified. To date, diabetes educators and midwives are the only health professionals who have accessed the web-based read only function of the CR (with possible reasons for discrepancies described above). This suggests opportunities to promote the CR more broadly to other groups of health professionals. Furthermore, respondents reported that CR reports and meetings were useful, with most users preferring the prospect of receiving reports rather than attending meetings. Reports that include data on process and outcome of care measures, as well as benchmarks for comparison have been recommended [17]. The CR reports currently include process and outcome measures, however benchmarking is restricted by limited comparative national data for Aboriginal women. With expansion of the DIP CR to another region of Australia, benchmarking between regions will be feasible, however detailed consultation with the Clinical Reference Group in each region is a necessary first step.

Common barriers for integrating CRs include difficulty with data entry (electronic versus paper based; and ensuring it is timely) and adequate funding [32]. These factors may limit use of the CR. Minimising time lag is critical to providing effective feedback to care providers and improving health care delivery [17]. This is a clear benefit of the CR over other epidemiological data sources such as the MDC, in that MDC data are not reported for 2–3 years. Furthermore, while paper-based data collection is more common, particularly in the establishment phase of CRs [32], electronic systems reduce chance of error and are more reliable than other systems [19]. CRs can be an effective resource when funded appropriately, with health care systems benefiting from their usage [14,17]. The DIP CR has received some funding for work thus far, however on-going funding remains a challenge and partners involved have always been mindful of long-term sustainability. Data entry remains a significant resource and capacity issue, and thus the number of variables was significantly reduced in 2014, in order to optimise sustainability. In addition, complete integration of the CR into current electronic medical records systems has not been feasible, due to limitations of current systems (despite available funding) but these systems have multiple roles beyond that of this CR. We continue to work closely with key stakeholders involved in change of current systems to improve CR integration, recognising the critical importance of ongoing stakeholder involvement to ensure this occurs [33].

Future directions for the CR are to include postpartum follow-up of women with GDM and type 2 diabetes (funded by a National Health and Medical Research Council Global Alliance of Chronic Diseases grant for a systems-based post-partum intervention using the CR). This may also create an incentive for health professionals to increase their usage of the CR. A potential longer term sustainability opportunity could be expanding the MDC to include the key additional variables of CR to the MDC, however timeliness of reporting would also need to be addressed. Recent funding also includes scale-up of the CR to another region of Australia with a high proportion of Aboriginal and Torres Strait Islander peoples. Expansion of the CR to further regions of Australia is also viable, particularly in light of the high rates and associated health outcomes among Aboriginal peoples [34].

### Limitations

This study had a number of limitations. First, the limited number of CR users restricts the generalizability of survey findings to a small cohort of diabetes educators and midwives. Furthermore, it is likely that these participants (and those who took part in focus groups) have been involved with the Partnership activities, highlighting a potential bias of these results. Second, some participants reported not knowing about the CR, yet completed questions associated with some knowledge of the CR. While the proportion of clinicians who responded to the survey is unknown, the number of health professionals working with women with DIP is small (estimated <300 in the NT); thus, this response is likely to be reasonably representative. Greater representation of Aboriginal Health Practitioners would have been of benefit considering their critical role in caring for Aboriginal women. Focus groups were broad in addressing a number of issues and did not focus specifically on the CR, thus thematic saturation was not necessarily reached. The results of the focus groups are likely to have been positively skewed based on the sample of participants (i.e. focus groups occurred after a Partnership event). However, a strength of this study was identifying reasons for non-use as obtained from a broad range of health professionals in the survey. Despite weaknesses in survey findings, triangulation of information from multiple data sources in this study provides valuable insights into current use and future directions of the CR. Furthermore, the first author was not involved in the implementation of the CR and maintained sufficient independence to report on this evaluation critically.

## Conclusions

In the context of improving outcomes for DIP in regions of Australia with a high proportion of Aboriginal women, the CR is contributing to improved understanding of epidemiology and disease burden. Prior to the establishment of the CR, the prevalence of DIP in the region appears to have been significantly under-estimated, particularly among Aboriginal women. The CR and related work of the DIP Partnership has contributed to significant increases in reported DIP prevalence, likely due to improved awareness, screening and reporting. This highlights that a CR should not be developed in isolation, rather alongside other efforts to reform health systems. However usage of the CR was limited in relation to individual patient care and there are opportunities for improvements. This study has highlighted important mechanisms for implementing a CR in a remote setting, with successes relating to stated aims in quality assurance and epidemiology. These learnings will be applied to expansion of the CR to other regions of Australia.

## Supporting information

S1 File NT DIP Partnership, Governance Structure, CR methods. (DOCX)

S2 File Survey. (PDF)

## Abbreviations

ADIPS: Australian Diabetes in Pregnancy Society
DIP: Diabetes in pregnancy
GDM: Gestational Diabetes Mellitus
IADPSG: International Association of the Diabetes and Pregnancy Study Groups
MDC: Midwives Data Collection
NT: Northern Territory
WHO: World Health Organisation
