# Supplementary material for: Implementation of a diabetes in pregnancy clinical register in a complex setting: Findings from a process evaluation
Source: PLoS One. 2017 Aug 4;12(8):e0179487. doi: 10.1371/journal.pone.0179487 (PMC5544201; doi:10.1371/journal.pone.0179487)
Supplement: S1 File — (DOCX) [file pone.0179487.s001.docx]

**Supporting Information**

The NT DIP Partnership aims to improve systems and services to improve outcomes for women with DIP, and their children. In 2010, partnership investigators commenced consultation with health care providers, regarding the DIP CR, which began operating in September 2011.

Governance Structure:

Clinical Register development and management was overseen by the DIP CR Working Group initially and then transferred to the Steering Committee (who receive feedback and clinical guidance from the Clinical Reference Group (CRG)) (Figure 1). The DIP CR working group was established in April 2011 with representatives from the following partners: NT Department of Health, Menzies School of Health Research, Aboriginal Medical Services Alliance of NT (AMSANT), Baker IDI Heart and Diabetes Institute and Healthy Living NT (HLNT). The working group completed the following:

1. Developed operating principles according to the principles of Australian Commission on Safety and Quality [1].
2. Defined variables for inclusion and developed policy for process of referrals.
3. Developed web-based read-only access for health professionals.
4. Formulated policies regarding CR access and auditing functions of users.
5. Developed processes for generating grouped reports for dissemination to clinicians and service providers.

From 2012, the working group functions were transferred to the NT DIP Steering and Management Groups. The Steering Group (Partnership chief investigators) meet quarterly and oversee CR management and conduct. The CR Management Group met quarterly (2012-2014) and included clinicians, health information systems analysts and Partnership investigators. Its purpose was to oversee day-to-day register management. From the end of 2014, the role of this group was performed directly by the CR regional managers (who report to the Steering Group). It was agreed that data on the CR will be used for clinical care and quality assurance. Applications may be made to the Steering Committee for publication of CR audits.

Inclusion criteria:

All women residing in NT of age 16 years and above with any type of DIP (type 1, type 2 and gestational diabetes) are eligible. In January 2015, an amendment was made to include pregnant women of all ages (with verbal parental/guardian consent for those under 16 years) as requested by the Clinical Reference Group. All participants are informed of the CR purpose and how to request removal. Verbal consent is obtained and documented at time of referral.

Access:

Read-only web based access is provided to health professionals involved in patient care on application. Access is via a password-secured link through the internet and associated documents such as referral forms, operating principles and information forms are located online [2].

Referral:

Women are referred from all areas of the NT. Referral is made by health professionals completing the standard template either by hand or electronically (can be auto-populated by two primary health care electronic medical record systems: Primary Care Information System and Communicare).

Data collection and quality assurance:

A standard referral and data collection template was created using the variables of the Australian Diabetes in Pregnancy Society National Audit Project [3]. The CRG reviewed the data collection template in 2014 and reduced the number of variables in order to optimise CR sustainability (number reduced from 99 to 71).

Core maternal and perinatal outcome data are collected and entered by appropriately trained hospital-based diabetes nurse educators and midwives. Data entry access is limited to ensure good quality data. It was agreed that data on the CR will be used for clinical care and quality assurance. Applications may be made to the Steering Committee for publication of CR audits.

Biannual grouped data reports are provided to clinicians for feedback on quality of care. The reports are presented at DIP CR regional meetings that facilitate discussion of results and implications for clinical management. A six monthly audit of CR users is conducted. Weekly reports by diabetes educators involved in the management of women with DIP are generated to enhance care coordination and antenatal follow-up of women.

*Figure 1.* Governance structure of NT DIP Clinical Register

**References:**

1. Australian Commission on Safety and Quality in Health Care: **Operating Principles and Technical Standards for Australian Clinical Quality Registries**. In*.* Sydney: Australian Commission on Safety and Quality in Health Care; 2008.

2. **NT Diabetes in Pregnancy Clinical Register (DPCR)** [<http://www.health.nt.gov.au/Chronic_Conditions/Diabetes_in_Pregnancy_Clinical_Register/index.aspx>]

3. Simmons D, Cheung NW, McINTYRE HD, Flack JR, Lagstrom J, Bond D, Johnson E, Wolmarans L, Wein P, Sinha AK: **The ADIPS pilot national diabetes in pregnancy audit project**. *Australian and New Zealand journal of obstetrics and gynaecology* 2007, **47**(3):198-206.
