# Supplementary material for: Implementation of a diabetes in pregnancy clinical register in a complex setting: Findings from a process evaluation
Source: PLoS One. 2017 Aug 4;12(8):e0179487. doi: 10.1371/journal.pone.0179487 (PMC5544201; doi:10.1371/journal.pone.0179487)
Supplement: S2 File — (PDF) [file pone.0179487.s002.pdf]

Consent

This survey is for Health Professionals involved in the management of pregnant women. The purpose of the survey is to assess Northern Territory health professionals' understanding and usage of the Clinical Register for diabetes in pregnancy. The results of the survey will provide information on the usefulness of the Clinical Register and will inform necessary improvements. Results will be reported grouped and de-identified to relevant professional and scientific bodies. In order to ensure security of the data, survey results will be downloaded and then deleted from the online electronic survey www.surveymonkey.com site and stored in a secure computer within Menzies School of Health facilities.

If you have questions about the survey please contact Menzies School of Health project officer Cherie Whitbread on 89468696 or cherie.whitbread@menzies.edu.au. If you have any concerns or complaints about the survey or project, you may contact the Secretary of the Human Research Ethics Committee of NT Department of Health and Menzies School of Health Research Phone 08 89468687 or ethics@menzies.edu.au.

We hope you are able to take a few minutes to participate in our survey. Your feedback is important.

1. Please indicate your consent in order to proceed with the survey:

- ◯ I agree
- ◯ I do not agree

Individual context

The following questions aim to collect some personal information about you.

2. What is your healthcare profession?

- Registered Nurse
- Registered Midwife
- General Practitioner
- Endocrinologist
- Obstetrician
- Dietitian
- Medical Practitioner (other, please specify below)
- Diabetes Educator
- Aboriginal and Torres Strait Islander Health Practitioner

Other (please specify)

3. What is your primary work place?

- General practice
- Health centre
- Hospital
- Other (please specify)

4. How long have you been in your current position?

5. In which region of the NT do you live?

- Central Australia
- Top End

6. Please nominate locality

- ◯ Remote
- ◯ Regional
- ◯ Urban

7. What age are you?

- ◯ 20-29
- ◯ 30-39
- ◯ 40-49
- ◯ 50-59
- ◯ 60-69
- ◯ 70+

8. What is your country of graduation?

Knowledge of the Clinical Register

The following questions aim to collect some information about what you already know about the register.

9. Have you ever heard of the NT Diabetes in Pregnancy Clinical Register?

- ◯ Yes
- ◯ No

If yes, how did you hear about it?

10. Have you been provided with any information about the NT DIP Clinical Register?

- ◯ Yes
- ◯ No (proceed to question 13)

11. If yes, what type of information have you been provided with?

12. Has this information been useful?

- ◯ Yes
- ◯ No

13. Has the purpose of the register been clearly outlined for you?

- ◯ Yes
- ◯ No

Knowledge of the Clinical Register for individual case management.

The following questions assess whether you know how to apply your knowledge of the Clinical Register in managing individual patients in day to day practice.

14. Do you know how to refer a woman to the NT DIP Clinical Register?

- ○ Yes
- ○ No (proceed to question 16)

15. How do you refer?

- ○ Electronic referral
- ○ Hand filled paper referral
- ○ Email
- ○ Fax
- ○ Other (please specify)

16. Health Professionals are able to apply for read only access to the Clinical Register. If you were to access the Clinical Register, what information would be useful for you to know about women who are currently pregnant or post-partum?
You can select more than one response.

- ☐ Past obstetric history
- ☐ Current DIP management
- ☐ Latest clinical review

Other, please specify:

17. Do you know how to apply for access to the clinical register?

- ○ Yes
- ○ No (proceed to question 19)

18. Have you applied for access?

◯ Yes

◯ No

If you have applied for access but never used it, why not?

19. Have you used the Clinical Register at any stage?

◯ Yes

◯ No (proceed to question 21)

Use of the Clinical Register for individual case management

The following questions assess whether you apply your knowledge of the Clinical Register to manage your patients in your clinical practice.

20. Please rate the following:

	Strongly agree	Agree	Can't say	Disagree	Strongly disagree
The Clinical Register is easy to access.	○	○	○	○	○
I always use the Clinical Register when caring for women with DIP.	○	○	○	○	○
Internet effects online accessibility.	○	○	○	○	○
Information is not always there in a timely manner.	○	○	○	○	○
I don't need to log in to the Clinical Register to view individual files because communication has improved between services and sectors.	○	○	○	○	○
Since 2012, shared electronic health records have improved.	○	○	○	○	○
Since 2012, my need to access the Clinical Register has decreased.	○	○	○	○	○
I feel more confident in the management of DIP that I don't need to access Clinical Register in individual care.	○	○	○	○	○
The NT DIP Partnership has improved education, orientation and guidelines.	○	○	○	○	○
Since commencement of the Clinical Register and NT DIP Partnership in 2012, communication has improved between sectors and services involved in DIP.	○	○	○	○	○

21. Do you think care-coordination has improved since 2012 with the implementation of the CR?

- ◯ Yes
- ◯ No

Please give reasons for both yes or no response.

22. Do you think that clinical registers are useful with assisting in providing individual clinical care for women with diabetes in pregnancy?

- ◯ Yes
- ◯ No (proceed to question 24)

* 23. What role do you see the NT DIP Clinical Register having in your own work place?

Value of the Clinical Register: reports and meetings

The following questions are to assess the usefulness of the meetings and reports related to the Clinical Register.

24. Prior to January 2016, did you receive any NT DIP Clinical Register reports?

- ◯ Yes
- ◯ No (proceed to question 26)

25. Do you find these useful?

- ◯ Yes
- ◯ No

26. Have you attended any regional meetings?

- ◯ Yes
- ◯ No (proceed to question 28)

27. Do you find these useful?

- ◯ Yes
- ◯ No

28. Would you like to be involved in:

	Yes	No
Attending regional meetings	◯	◯
Receiving reports	◯	◯

29. Have any changes occurred in your workplace as a result of the clinical register reports or regional meetings?

- ◯ Yes
- ◯ No (proceed to question 31)

30. Please rate the following. The Clinical Register has:

	Strongly agree	Agree	Neither agree nor disagree	Disagree	Strongly disagree	Don't know
Increased communication.	◯	◯	◯	◯	◯	◯
Improved understanding the recommended clinical care required for women with diabetes in pregnancy.	◯	◯	◯	◯	◯	◯
Improved awareness of early detection of diabetes in pregnancy.	◯	◯	◯	◯	◯	◯
Improved awareness of pre-pregnancy planning & contraception.	◯	◯	◯	◯	◯	◯
Improved awareness of who to contact in regards to women with DIP.	◯	◯	◯	◯	◯	◯
Improved awareness of how many women in the NT have DIP.	◯	◯	◯	◯	◯	◯

Please describe any other benefits of the Clinical Register:

Improvements to the Clinical Register

We would like to hear from you about how to improve the Clinical Register.

31. From your experience, in what ways could the Clinical Register be improved?

32. Any other comments?
